# Supplementary material for: Chromothripsis during telomere crisis is independent of NHEJ, and consistent with a replicative origin
Source: Genome Res. 2019 May;29(5):737–49. doi: 10.1101/gr.240705.118 (PMC6499312; doi:10.1101/gr.240705.118)
Supplement: Supplemental Material [file supp_gr.240705.118_Supplemental_file_1.zip › contigs/annotated_contigs/DB51/contig.2.DB51_length_604_mean_cov_7.03642384106.docx]

**DB51_length_604_mean_cov_7.03642384106**

```
AACGGGAGATGGAAGAGCATTTGGCAAGCAGTCAGGAAACTGGAGTTCTTATGCTAGGACCCTGACACTGAATGGTCATATATTTTGGG
 >chr18:68567781-68568054 - E=1e-152
AAAGTCTGTAAGAACCCTGGATCTCCTTATGTGAAAGTAGTTCAGCTAACTGCATCATTTCCAAGACTTAAGTTCCAAAATTTTGTGAA

CGTCTATGTGGAAGCTTGTATATGTAAGATAACTTAATTCTTAGGAGAGTGTCTCAGTTTTTTTCCTGGGGCTGCCATAACAAAGCACT

ATAAAC|AA|GTATGATTCCAAAAATTGCTTCTCTTTCAAGTAGTTACACCTGTGGATACAATGTCTATCACCATAACTTCGACTCCTG
         >chr6:134698001-134698330 - E=3e-186
AGCAAAATGAGAGTGATCATAAGTGGAATCCCTAGTAGGTATATCAATCAGGGCCCCAACAGGAAGCAAGTGGCATACTCCAGGAGGGT

TGAGTAAAGGGACTGTTGATAGGGTATAGGCAGATGTGGGAAAACCACAAAGAAGAGTGACATGCTGGGGCTAGTCACAGCAGATGTTA

CAACCCTGAGGTGTAAAGGAGCAAGAGGAGAGAGTGTGGAAAGGGTCACCTTGAGAGGAGTTATGGTCTTTG
```
